# Cohort Profile Update: The Doetinchem Cohort Study 1987–2028––capturing four decades of health and disease

**DOI:** 10.1093/ije/dyag120

**Published:** 2026-07-21

**Authors:** H Susan J Picavet, M Liset Rietman, Anneke Blokstra, W M Monique Verschuren

**Affiliations:** Centre for Prevention, Lifestyle and Health, National Institute for Public Health and the Environment, Bilthoven, The Netherlands; Centre for Prevention, Lifestyle and Health, National Institute for Public Health and the Environment, Bilthoven, The Netherlands; Centre for Prevention, Lifestyle and Health, National Institute for Public Health and the Environment, Bilthoven, The Netherlands; Centre for Prevention, Lifestyle and Health, National Institute for Public Health and the Environment, Bilthoven, The Netherlands; Julius Center for Health Sciences and Primary Care, University Medical Center Utrecht, Utrecht, The Netherlands

Key Features

- Originating from a monitoring study on cardiovascular disease risk factors, the Doetinchem Cohort Study has been expanded to a long-running study on lifestyle and other determinants of health, functioning, and disease over the life course.
- Starting with a random sample of men and women aged 20–59 years, two-thirds of those measured at baseline were re-invited to participate in the cohort. The response rates in Waves 2–8 were on average 78%. In Wave 7 (2018–23) and Wave 8 (2024–8), the numbers were increased by inviting part of the original one-third who were not enrolled in the cohort.
- Strengths are the continuation of many health and health-related measures over 35–40 years, making the study of long-term individual changes possible and the development of research on ‘omics’.
- We welcome collaboration for using the data; please contact the team of the Doetinchem Cohort Study at SAG@rivm.nl.

## The original cohort

**Figure 1:**
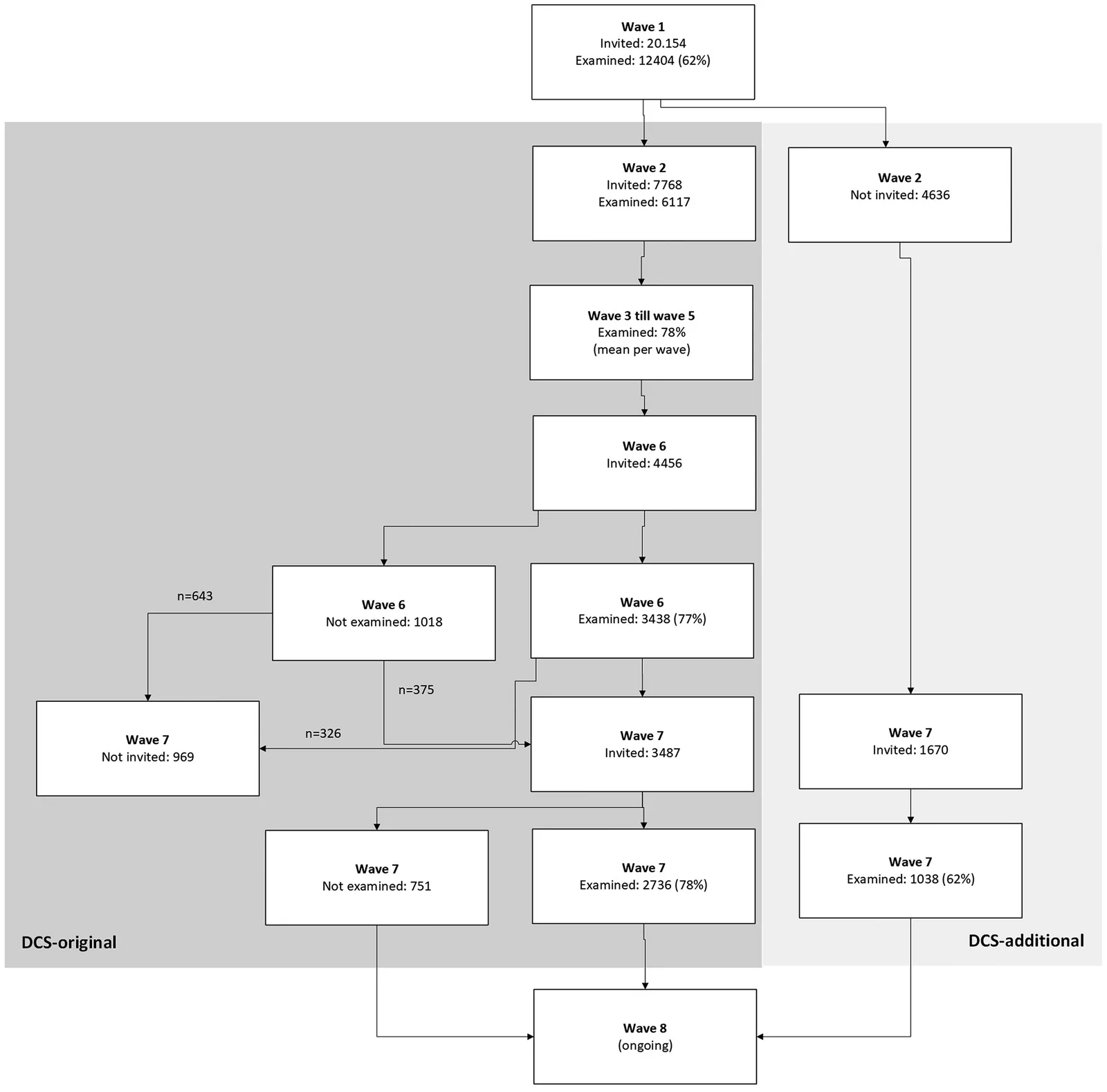
Flow chart of the DCS.

The Doetinchem Cohort Study (DCS) was started as a monitoring study on cardiovascular health in 1987–91 based on an age–sex stratified sample of the population register of inhabitants of Doetinchem, with equal numbers of men and women and equal numbers in 10-year age categories: 20–29, 30–39, 40–49, and 50–59 years, *n* = 12 404. The cohort was established by re-inviting a two-thirds sample (*n* = 7768) of those who were measured during Wave 1 for a second measurement in 1993–7, followed by a third in 1998–2002 [1], a fourth in 2003–7, a fifth in 2008–12, and a sixth in 2013–17 [2], including 3438 participants (Fig. 1).

## What is the reason for the new data collection?

The continuation of the data collection makes it possible to study health, lifestyle, and ageing over an even larger part of the life course. The initial general population sample, both men and women aged between 20 and 59 years, has been followed for almost 35 years and aged, during Wave 8, between 56 and 95 years. In addition, new advances in technologies have opened up opportunities for research, e.g. in genetics and metabolomics. We added datacollection on infectious diseases and inflammation and expanded the collaborations, making meta-analyses possible [3–6] and large-scale consortia, such as on the prevention of dementia Netherland Consortium of Dementia Cohorts (NCDC) and Boosting Insights into risk Reduction of Dementia (BIRD-NL consortium), on lifetime depression (BIONIC) [7], and on research on health and the menopause (Dutch MenoPause Consortium).

We work with 11 Dutch cohorts to improve the collaboration in creating an infrastructure for this: the Netherlands Cohort Consortium (NCC) [8].

## What will be the new areas of research?

For this second update, the new areas are:

- biomarkers of ageing [metabolomics, inflammation markers, and (in a subsample) ptau and beta-amyloid], characterizing profiles associated with healthy ageing;
- long-term individual change in lifestyle and health, mental health;
- the role of lifestyle and health before, around, and after menopause;
- broadening the scope with studies on infectious diseases, a.o. immunological response to COVID-19 vaccination, and the evaluation of willingness to get vaccinated.

## Who is in the cohort?

Wave 7 was carried out in 2018–23 and Wave 8 is ongoing (2024–8). Participants included in the original cohort have now been measured up to eight times (*n* = 2736 of the original cohort in Wave 7). The response rates are high (∼78%) but of course there is attrition due to mortality, movements of participants abroad, or the active or passive withdrawal of participants. We excluded those who did not participate in three consecutive waves. Active withdrawal refers to those who indicate not wanting to be invited again and passive withdrawal refers to those who did not respond to the invitation.

During Wave 7, we also invited part of the third who were not invited for Wave 2, to increase the numbers (*n* = 1670). Those participants were thus measured in Wave 1, Wave 7, and Wave 8 (Table 1 and Fig. 1) and are referred to as DCS-additional, in contrast to DCS-original (*n* = 1038 in Wave 7). In total, Wave 7 consisted of 3774 participants comprising men and women aged between 51 and 90 years.

**Table 1:** Overview of number of measurement waves of the DCS.

	Men	Women	Total
Participated in seven waves	1079	1210	2289
Participated in 4–6 waves	1046	1149	2195
Participated in 2 or 3 waves^a^	1289	1539	2828
Total number of participants^a^	3414	3898	7312
Cohort members deceased since Wave 2^b^	1215	1131	2346

a Including the participants who were invited to participate in Waves 1 and 7 only.

b From those who were invited for Wave 2 (*n* = 7768); unknown for participants who did not give permission for linkage to the municipal registration (*n* = 169).

A total of 2289 participants attended all waves. In addition, 2195 men and women participated in four to six waves and 2828 participated in two or three waves (this includes DCS-additional).

As of July 2025, 2346 of the 7768 DCS-original participants (30%) had died.

## What has been measured?

From the start, standard themes and measurements related to chronic diseases were included with an emphasis on cardiovascular diseases, diabetes, musculoskeletal disorders, respiratory diseases, and cancer (Table 2). These have all been continued during later waves, including the collection of blood. From Wave 4 onwards, we expanded the measurement protocol and the possibility of side studies has also been further exploited. These new measurements are described below, as well as information on the biobank and the side studies provided.

**Table 2:** Overview of themes measured in the DCS: questionnaires and measurements during physical examination.

	Wave							
	1	2	3	4	5	6	7	8
**Background characteristics**								
Age, sex, marital status, work status, household characteristics	x	x	x	x	x	x	x	x
Educational level (a)	x	x	x	x			x	
(Historical) shift work, societal participation, birthweight						x		
**Lifestyle factors**								
Smoking (current and history)	x	x	x	x	x	x	x	x
Physical activity by questionnaire (b)	x	x	x	x	x	x	x	x
Physical activity by Actigraph						x	x	x
Sedentary time		x	x	x	x	x	x	x
Alcohol consumption	x	x	x	x	x	x	x	x
Sleep (hours)	x	x	x	x	x	x	x	x
Sleep (quality)	x					x	x	x
Chronotype						x	x	
**Biological factors**								
Standing height, weight, blood pressure (arm)	x	x	x	x	x	x	x	x
Blood pressure (also ankle)					x	x	x	x
Lung function (c), waist and hip circumference		x	x	x	x	x	x	x
For women: reproductive history and additional characteristics on menstruation, contraception use,	x	x	x	x	x	x	x	x
heel bone mass					x	x		
Advanced glycation end products in the skin						x	x	x
Circumference of the arm							x	x
Body composition using bioimpedance							x	x
**Functioning**								
Quality of life (SF36)		x	x	x	x	x	x	x
Cognitive test battery		x	x	x	x	x	x	x
Hearing and eyesight issues				x	x	x	x	x
Hand-grip strength					x	x	x	x
Balance test, chair test					x	x	x	x
Walking speed							x	x
**Psychosocial aspects**								
Depressive symptoms, loneliness					x	x	x	x
Health literacy		x				x	x	x
Perception of living environment (in particular, possibilities for recreational physical activity)						x	x	
Coping and personality characteristics (e.g. John Henryism)	x	x					x	
Social support					x	x	x	x
**Chronic diseases**								
Diabetes	x	x	x	x	x	x	x	x
Cardiovascular disease (stroke, heart disease)	x	x	x	x	x	x	x	x
Claudication		x	x	x	x	x	x	
Migraine, musculoskeletal pain, cancer, asthma and Cronic Obstructive Pulmonary Disease (COPD) (complaints)		x	x	x	x	x	x	x
Asthma and COPD (disease)					x	x	x	x
(History of) fractures					x	x	x	x
List of chronic conditions (including e.g. incontinence, falling, osteoarthritis)					x	x	x	x
**Divers**								
Family history on diseases (e.g. heart diseases, fractures, dementia, menopause)					x	x	x	x
Oral health (teeth and gum)						x	x	x
(Historical) exposure to sound pollution, contact with (small) children, consumption of tap water						x		
**Healthcare**								
Use of healthcare services, self-management, giving and receiving informal care						x	x	x
Willingness to get vaccinated against influenza, pneumococcal disease, pertussis, and herpes zoster						x	x	

(x) measured in the same respondents: CXCL9/MIG, IFNg, CCL11/Eotaxin, CXCL1/Gro-alpha, IL-1a, IL-6, IL-10, IL-18, CCL2/MCP-1, CXCL10/IP10, CXCL8/IL-8, CD163, YKL40/CHI3L1, CXCL11/I-TAC, GDF15, Gp130, IL6RA, Leptin, MMP12 (elastase), TRAIL, TNF RI/TNFRSF1A, Adiponectine, C-reactive Protein (CRP), CD14, hsIL-6, hsIFNg.

### New measurements during the physical examination

Since Wave 6, the physical examination has been extended with measurements of arm circumference, normal walking speed (5 metres), body composition using bioimpedance, and a collection of a faeces sample for the microbiome.

### New themes in questionnaires

The main themes for which the questionnaires have been extended are:

- willingness to be vaccinated against influenza, pneumococcal disease, pertussis, and herpes zoster [9];
- coping and personality characteristics: John Henryism [10];
- a newly developed online Food Frequency Questionaire (FFQ) [11].

### Biobank samples and measures

A total of 25 ml of non-fasting venous blood is collected in every wave by using three types of vacutainers: NaF, serum, and Ethylenediaminetetraacetic acid (EDTA). Samples are centrifuged and aliquoted in 25 cryovial tubes, including plasma, serum, erythrocytes, and buffy coat, and stored at −80°C. Blood samples are available for all participants for all waves, though there are some differences in each measurement wave regarding the storage conditions or the number of tubes.

A spot urine sample was taken and stored at −80°C, after testing for urinary tract infection. In Waves 7 and 8, we also collected stool samples.

Overviews of all omics (available and planned) are presented in Table 3.

**Table 3:** Overview of themes measured in the DCS: biomaterial and biochemical markers.

	Wave							
	1	2	3	4	5	6	7	8
**Material: Non-fasting blood**								
Plasma (Ethylenediaminetetraacetic acid (EDTA))	x	x	x	x	x	x	x	x
Plasma (sodium floride (in Dutch NaF))	x	x	x	x	x	x	x	x
Plasma (kalium EDTA)			x	x	x	x	x	
Serum		x	x	x	x	x	x	x
Buffy coat	x	x	x	x	x	x	x	x
Erythrocytes	x	x	x	x	x	x	x	x
**Material: Urine (spot)**					x	x	x	x
**Material: Faeces**							x	x
Microbiome (16S, *n* = 1000)							x	
**Biochemical markers (regular)**								
Total and HDL cholesterol	x	x	x	x	x	x	x	x
Blood glucose (non-fasting)		x	x	x	x	x	x	x
Creatinine					x	x	x	x
**Biochemical markers (project-based)**								
Albumin/alat/GGT/create/triglycerides/urea/C-reactive Protein (CRP)/cystatine C		x	x	x	x			
Metabolomics (Nightingale platform)				x		x		
Anti-Mullarian Hormone (women only)	x	x	x	x	x			
Single-nucleotide polymorphisms (GSA array, Illumina, *n*=∼ 5000)					x			
Immune markers (a)		x		x		x		
Biochemical markers (planned)								
pTau-217 (*n* = 400)				x		x		
Proteomics (somalogic) in *n* = 363 (of whom 78% died at different ages)	x	x	x	x	x	x	x	

(a) measured in the same respondents (n=1998): CXCL9/MIG, IFNg, CCL11/Eotaxin, CXCL1/Gro-alpha, IL-1a, IL-6, IL-10, IL-18, CCL2/MCP-1, CXCL10/IP10, CXCL8/IL-8, CD163, YKL40/CHI3L1, CXCL11/I-TAC, GDF15, Gp130, IL6RA, Leptin, MMP12 (elastase), TRAIL, TNF RI/TNFRSF1A, Adiponectine, C-Reactive Protein (CRP), CD14, hsIL-6, hsIFNg. HDL, high-density lipoprotein; gamma-glutamyltransferase, GGT.

### Side studies

Lifetime depression: in 2017, 2750 out of 4080 active participants filled in an additional postal questionnaire on lifetime depression [12] as part of the BIONIC consortium on the genetics of depression [7].

Immunosenescence: a number of cellular and molecular immune markers, including peripheral blood mononuclear cells and CMV infection, were measured in additional blood samples for 300 men and women aged 60–85 years, stratified by a high, average, or low frailty score. This was used to study which aspects of the ageing immune system are most relevant in the process of reaching old age in good health [13].

Vaccination response pneumococcal disease: from the autumn of 2020 onwards, vaccination against pneumococcal disease became part of the Dutch vaccination programme, starting with those aged 73–80 years. Among the participants of the DCS in this age group, the immune response was studied by using additional assessed blood and saliva measures before and after vaccination (*n* = 190).

Response to COVID-19 vaccination: all active participants of the DCS were approached for a study on their response to COVID-19 vaccination. For 1374 participants, we measured the IgG antibody concentrations in serum to SARS-CoV-2 Spike protein (S1) and Nucleoprotein (N) before and several times after vaccination [14].

Biomonitoring of cadmium and lead: in order to contribute to the monitoring of exposure to potentially harmful chemicals, additional blood and urine samples from 336 participants were assessed in order to measure cadmium and lead [15].

Enrichment through registers included:

- Environmental exposures: the DCS is part of the Geoscience and Health Cohort Consortium (GECCO) in the Netherlands, which aims to centralize all available geodata and the linkage of these data to individual-level data from longitudinal cohort studies to enable large-scale epidemiological research on the impact of the environment on public health in the Netherlands [16].
- Health and demographic data: linkage with registers for additional socio-demographic characteristics (such as income) with healthcare registers (e.g. hospital-discharge diagnosis, GP data, healthcare costs, and medication use) and cause-of-death registers through linkage with Statistics Netherlands (CBS).

## What has it found? Key findings and publications

### Long-term changes in lifestyle

For several health-related lifestyle factors (physical activity, weight, smoking, sleep, and alcohol consumption), we studied the population trends and individual changes over a period of ≤30 years (a.o. [17, 18]). The population trends over 30 years in physical inactivity and ‘unhealthy’ alcohol consumption were stable; overweight and unhealthy sleep prevalence increased and smoking prevalence decreased. The proportion of the population being healthy on all five lifestyle factors declined from 17% in Wave 1 to 11% in Wave 6. Underlying these trends, a dynamic pattern of changes at the individual level was seen: sleep duration and physical-activity levels changed in almost half of the individuals, body mass index (BMI) and alcohol consumption changed in one-third, and smoking changed in one-quarter. This shows that population trends do not give an insight into change at the individual level. In order to be able to gauge the potential for a change in health-related lifestyles, it is important to take changes at the individual level into account. With the increased prevalence of overweight, we also showed that younger generations had obesity at an earlier age but did not reach higher levels at midlife and beyond. However, this does still result in a higher lifetime burden of obesity [19].

### Long-term changes in health

For several health indices, we explored the way in which the development of health over the life course and while ageing can be depicted. For pain, we found that a substantial part of the population reported pain over a long period of their life course [20]. Long‐term trajectories of pain may reflect phenotypes that may be relevant to take into account in pain management. Several risk factors, such as short sleep duration, smoking, obesity, and poor perceived or mental health, were associated with these trajectories and tackling these may contribute to the prevention of pain over the life course. We also studied the long-term changes in the Healthy Aging Index (HAI) [21]—an index of physiological ageing, combining levels of systolic blood pressure, non-fasting plasma glucose levels, global cognitive functioning, plasma creatinine levels, and lung functioning. Using latent class mixture modelling, we found one HAI trajectory for women and two for men, labelled ‘gradual’ ageing (76%) and ‘early’ ageing (24%). At ages of 30–70 years, ‘early’-ageing men had 5 fewer healthy years compared with ‘gradual’-ageing men. Long-term patterns in vitality, as measured every wave by using the SF-36 vitality scale, were studied via both predefined and data-driven methods [22]. The predefined patterns were: persistent good, persistent poor, worsening, improving, and varying vitality. Data-driven analysis revealed patterns similar to those of the predefined method with ‘varying vitality’ split into two variants. There was fair agreement between the approaches (Cramér’s V: 0.49). Most participants exhibited persistent good vitality: 61% in the predefined and 78% in the data-driven approach, and mental health was a strong driver of the patterns.

### Cognitive decline

When studying cognitive decline, we observed that women perform better for all cognitive domains compared with men but also show faster cognitive decline with ageing [23]. Better adherence to dietary guidelines (highest tertile) was associated with better cognitive performance and slower cognitive decline (compared with the lowest and middle tertiles) [24]. The metabolomic profiles associated with cognitive function and decline differed between men and women: in men, mostly markers of glucose metabolism and amino acids were associated with cognitive function whereas, in women, this was the case for markers of lipid metabolism and inflammation [25]. Obesity was associated with worse cognitive functioning compared with healthy weight for both men and women [26].

### Biomarkers research

Among the women, we studied Anti-Mullarian Hormone (AMH) concentrations over time and showed that the AMH-decline rate does not improve the prediction of menopause. Based on the low discriminative ability and underestimation of the risk of early menopause, the use of AMH as a screening method for the timing of menopause still does not seem feasible [27].

We studied the long-term trajectories of a number of biomarkers in relation to cognitive frailty [28]: total and high-density lipoprotein (HDL) cholesterol, triglycerides, alanine aminotransferase, gamma-glutamyltransferase (GGT), high-sensitivity C-Reactive Protein (CRP), albumin, uric acid, cystatin C, and creatinine. Of those, only the trajectories of total cholesterol, GGT, and urea seem to be associated with cognitive frailty, and only among women. We do not yet consider any of the studied biomarkers to be promising biomarkers for cognitive frailty. We also studied the potential of relatively new health markers, such as advanced glycosylated end products (AGEs). We showed that AGEs were associated with frailty. Both AGEs and metabolic markers (MetaboHealth) were independently associated with frailty, implying that they each capture distinct characteristics of the ageing process [29].

### Miscellaneous

We studied sex differences in healthy ageing, showing that men are doing better on functional health [30] while women were doing better on cognition [23]. We also studied gender by using a scale of masculine-connoted aspects on the prevalence of chronic conditions. Masculine gender was associated with a lower prevalence of chronic health problems in both men and women but, particularly in men, a higher masculinity score was associated with better health [31].

Some key measurements (blood pressure, blood lipids, body weight, smoking) of the participants in Wave 7 are presented in Table 4.

**Table 4:** Characteristics of the participants in Wave 7 of the DCS: DCS-original comprising those who were invited from Wave 1 onwards and DCS-additional comprising those who participated in Wave 1 and were invited again from Wave 7 onwards.

	Total (*N* = 3774)	DCS-original (*n* = 2736)	DCS-additional (*n* = 1038)
Age (years) [mean (SD)]	67.8 (8.6)	68.2 (8.5)	66.9 (8.7)
50–60 (%)	25	23	31
61–70 (%)	41	41	39
71–80 (%)	27	29	23
81+ (%)	8	8	7
Men (%)	47	48	46
Married/living together (%)	76	77	75
Paid job (%)	37	34	43
Smoking cigarettes (%)	7	8	6
Consuming alcohol (≥1/week) (%)	58	58	58
Being physically active^a^ (%)	65	65	65
BMI [mean (SD)]	26.7 (4.4)	26.7 (4.4)	26.4 (4.4)
Overweight (BMI 25–30 kg/m^2^) (%)	44	44	43
Obese (BMI ≥ 30 kg/m^2^) (%)	19	19	17
Waist circumference (cm) (%)	97	97	96
Abdominal overweight^b^ (%)	27	27	26
Abdominal obesity^c^ (%)	56	56	54
Diastolic blood pressure (mmHg) [mean (SD)]	77 (9)	77 (9)	77 (9)
Systolic blood pressure (mmHg) [mean (SD)]	132 (17)	132 (17)	133 (17)
Hypertension^d^ (%)	51	50	51
Total cholesterol (mmol/l) [mean (SD)]	5.3 (1.1)	5.2 (1.1)	5.3 (1.0)
HDL cholesterol (mmol/l) [mean (SD)]	1.5 (0.4)	1.4 (0.4)	1.5 (0.4)
Hypercholesterolemia^e^ (%)	38	38	36
Self-perceived health (≥ good) (%)	85	85	85
Myocardial infarction (ever) (%)	5	5	5
Stroke (ever) (%)	4	4	3
Diabetes^f^ (%)	8	9	7
Cancer (ever) (%)	16	15	17

a According to the Dutch recommended levels for adults (i.e. >3.5 h per week).

b Women: waist circumference 80–88 cm; men: waist circumference 94–102 cm.

c Women: waist circumference ≥ 88 cm; men: waist circumference ≥ 102 cm.

d Systolic blood pressure ≥ 140 mmHg and/or diastolic blood pressure ≥ 90 mmHg or and/or using blood-pressure-lowering medication.

e Total cholesterol ≥ 6.5 mmol/l and/or use of cholesterol-lowering medication.

f Self-reported diabetes and/or (non-fasting) glucose ≥ 11.1 mmol/l.

## What are the main strengths and weaknesses?

The DCS has several key strengths: (i) long-term follow-up of a randomly selected population-based sample covering a broad age range; (ii) detailed characterization of lifestyle factors (including dietary intake), health status, and disease, with repeated assessments of lung function, cognitive performance, and the collection of biological materials—including an extensive biobank with DNA samples at each wave; (iii) linkage to multiple external registries, such as cause-of-death, hospital-discharge, pharmacy, and cancer registries; and (iv) the integration of various ancillary sub-studies. The primary limitations of the study are as follows: (i) the cohort size is insufficient for the analysis of rare health conditions; (ii) there is a lack of representation from ethnic-minority subgroups; (iii) financial constraints have precluded the inclusion of new cohorts from younger age groups; and (iv) the presence of selection bias. It should be noted that, as is common in long-term cohort studies, there is a tendency for the sample to become enriched with individuals who are healthier and more highly educated.

The main challenges that we face in carrying out the study refer to the attrition and financing of the study. It is important to keep participants in the study even when they become frail. However, participating in the physical examination and filling out questionnaires become bigger challenges with the longer follow-up of the study, and participants do not like to notice that they are doing worse compared with 5 years previously, which is especially true in the case of the cognitive tests.

We are in the privileged position to have already carried out this study for >30 years. However, continuation of the study with an eighth measurement wave, maintenance of the biobank with samples from all previous waves, and the more complex organization regarding carrying out the study with increasingly expanding topics, as well as finding funding for the harvesting of these rich data, remain challenges.

## Can I get hold of the data? Where can I find out more?

We welcome collaboration with other researchers. The data of the DCS cannot be placed in a public repository due to legal and ethical constraints. The participants’ informed consent did not include consent to public availability of the data. Several types of collaboration to explore the data are possible; please contact the DCS via our Scientific Advisory Group (SAG@rivm.nl).

## Ethics approval

The study was conducted according to the principles of the World Medical Association Declaration of Helsinki and its amendments since 1964 and in accordance with the Medical Research Involving Human Subject Act (WMO). Approval was given on 31 December 2007 for Wave 5 and on 24 January 2013 for Wave 6 by the METC of University Medical Centre of Utrecht (UMCU) with protocol number 7/233 (and Central Committee on Research Involving Human Subjects (in Dutch CCMO)_n number 19158.041.07). The approval for Wave 7 was given on 131 March 2018 with protocol number 17–869 (and CCMO number NL63779.041.17). The data of approval for Wave 8 was 20 November 2023 (with number 23–169/X-A and CCMO number NL84982.041.23) including the biobank with number 23–184. The name of the METC of UMCU has changed to NedMec.
